# Assessment of Nutritional Risk Representations in Publicly Available Data in Saudi Arabia: A Triangulated Scoping Study

**DOI:** 10.3390/nu18172888

**Published:** 2026-09-03

**Authors:** Reham I. Alagal

**Affiliations:** Department of Health Sciences, College of Health and Rehabilitation Sciences, Princess Nourah Bint Abdulrahman University, P.O. Box 84428, Riyadh 11671, Saudi Arabia; rialagal@pnu.edu.sa

**Keywords:** nutrition surveillance, Saudi Arabia, food availability, dietary consumption, biomarkers, food balance sheets, fruit and vegetable consumption, low haemoglobin, hypercholesterolaemia

## Abstract

*Background:* Saudi Arabia maintains official statistics on its food security, in addition to conducting surveys on health parameters and performing national health assessments. Also, several academic papers on dietary patterns are published every year. Many published documents analyze data on food availability, self-reported eating habits, and biomarker-based health risks. However, individually, they represent distinct aspects of nutrition. Hence, this study was undertaken to examine publicly accessible nutrition-related evidence in Saudi Arabia and assess whether existing public food and nutrition datasets consistently align with the biological indicators of nutritional risk. *Methods:* Searches were conducted using defined criteria in publicly available Saudi and international sources published or updated between 2005 and 2025. These were identified through official reports, global food databases, WHO-aligned surveys, and peer-reviewed articles, with searches conducted and updated during June–July 2026. Sources were classified by representativeness, recency, measurement validity, accessibility, and analytical role. The primary outcome was the completeness and interpretability of public evidence streams rather than a pooled prevalence estimate. *Results:* SHIS 2013 remains the top public national dietary intake dataset, which shows that only 5.2% and 7.5% of people met their fruit and vegetable intake recommendations, respectively. GASTAT Health Determinants Statistics 2024 indicated that only 10.2% of people aged 15 years and above ate five or more portions of fruits and vegetables daily, yet GASTAT Food Security Statistics 2024 indicated significant per capita availability of rice, milk, poultry, eggs, red meat, onions, tomatoes, and dates. High levels of biological risk were evident in WHS 2019 biomarkers; for example, 42.6% of people had hypercholesterolemia and 50% had low hemoglobin according to the WHS 2019 indicator definition. Recent studies provided additional data on beverages, sugars, dietary habits of adolescents, and diet quality but lacked national food-wide intake data. *Conclusions:* Public Saudi nutrition data provide complementary but non-equivalent information; indicators show low dietary adequacy and high biomarker-defined risk, supporting the need for integrated individual-level nutrition surveillance. In the future, Saudi national nutrition surveillance must focus on individual-level intake data and biomarker-based measurements.

## 1. Introduction

Currently, nutrition surveillance largely depends on a growing combination of administrative statistics, food balance sheets, household surveys, individual dietary surveys, modeled datasets, biomarker surveys, etc. [1,2,3,4]. These sources complement each other but are not interchangeable [3,4]. For example, food availability data reflects the available food nationwide, whereas a dietary survey captures what individuals say they are consuming. Biomarkers, on the other hand, are indicators of the biological risks or results associated with a diet, disease, genetics, or social factors [1,2,3,4].

Efforts undertaken by the Kingdom of Saudi Arabia (KSA) demonstrate some of the gaps that can occur in nutritional surveillance. While experiencing rapid modernization, the country has implemented many policies to prevent non-communicable diseases (NCDs) related to diet, and it has invested considerably in its food system [5]. However, detailed, nationally representative public data on dietary intake are still lacking. Information collected in the 2013 Saudi Health Interview Survey (SHIS) remains the country’s most comprehensive publicly available evidence [6]. The 2019 World Health Survey may have yielded more robust biomarker data, but its dietary component is less comprehensive [7]. Recent GASTAT reports [8,9] include health determinant and food security data, but they are largely self-reported or average measures, not the actual nutrition of individuals.

Some significant developments identified in the scoping search include the probabilistic National Nutrition Consumption Model (NNCM) [10], the Saudi Healthy Eating Index [11], and the Saudi Healthy Plate 2024 [12]. These measures are useful for modeling and forming guidelines, but they also point to the problem at hand, for example, the lack of publicly available individual-level intake data from a national sample linked to objective biomarkers. Hence, in this work, we opted for a triangulation scoping study, where triangulation refers to the structured comparison of three non-equivalent public evidence streams—i.e., food availability statistics, self-reported dietary indicators, and biomarker-defined risk indicators—to assess whether current public data systems provide complementary or incomplete representations of nutritional risk, without treating these indicators as directly interchangeable, statistically poolable, or causally linked at the individual level. The present study differs from a narrative review because it uses predefined source categories, explicit eligibility criteria, structured extraction, and source appraisal to map what public Saudi nutrition evidence can and cannot support. It also differs from a simple evidence map because it operationally triangulates three non-equivalent evidence streams—food availability, self-reported dietary indicators, and measured biomarkers—to evaluate the interpretability and limitations of the current public nutrition surveillance ecosystem. This study addresses food and nutrition concerns, seeking to answer the following question: What publicly available evidence exists on food availability, dietary adequacy, and biomarker-defined nutritional or cardiometabolic risk in Saudi Arabia, and how should these non-equivalent evidence streams be interpreted for nutrition surveillance?

## 2. Methods

### 2.1. Study Design

This was a triangulation scoping study of publicly available data. The analysis was intended to identify and synthesize public nutrition evidence specifically for Saudi Arabia from official reports, global databases, and peer-reviewed publications. The study followed scoping-review principles consistent with PRISMA-ScR/JBI-style reporting by defining a research question, eligibility criteria, source categories, a search strategy, a screening process, extraction fields, and a synthesis approach. A meta-analysis was not performed because the data used included sources that differed substantially in design, population, dietary definitions, biomarker measurement, and availability of variance estimates. Future work could apply meta-analytic methods if more harmonized data become available.

### 2.2. Review Period and Search Dates

Sources published or updated between 2005 and 2025 were eligible. Searches were conducted on 21 June 2026 and again on 5 July 2026 before manuscript submission. The final search re-run date and screening documentation, including record counts, are provided in Supplementary Table S1 and Supplementary Figure S1.

### 2.3. Data Sources and Search Strategy

Searches of PubMed/PubMed Central (PMC), Saudi government portals, FAO/WHO platforms [1,2], materials connected to the Global Dietary Database [13], publisher pages, and accessible abstracts were conducted for the period of 2005 to 2025 used to find publicly available sources. The exact search keywords were saved for manual replication before journal submission; a full direct database export from Scopus and Google Scholar was not possible in this setting. The searches of public data were conducted on 21st June 2026 and again on 5th July 2026 before manuscript submission. These searches were carried out for the period between 2005 and 2025. The database names, search strings, and search results are shown in Table 1. Additionally, Supplementary Table S1 provides the database/source, search dates/updates, review period, and Boolean search string/retrieval approach. In total, 1190 data sources were identified: 1186 from PubMed, 2 from GSTAT Publications, and 2 from WHO/MoH reports (Supplementary Figure S1).

### 2.4. Screening and Duplicate Handling

Titles, abstracts, official report summaries, data tables, and full-text sources were screened in stages. Duplicate records were removed manually by comparing the title, author, year, DOI, source organization, and dataset identity using the spreadsheet “duplicate command”. Upon screening, 279 records were found to be duplicates and hence removed. After removing duplicates, 911 records were assessed based on eligibility criteria (Supplementary Figure S1).

### 2.5. Eligibility Criteria and Source Classification

The data sources were classified into three categories: publicly available, nationally representative, and contextual. Data sources that could be accessed without restricted institutional permission, data-use approval, or individual-level data application were considered publicly available sources; surveys and official statistical outputs designed to represent the Saudi national or resident population through national sampling frames, regional coverage, or official weighting procedures were considered nationally representative sources; and sources that informed interpretation but were not nationally representative, not food-wide, not biomarker-linked, or focused on a specific subgroup, region, food category, or dietary behavior were considered contextual evidence.

Sources that offered nutrition, diet, food availability, health determinants, biomarkers, or diet-related NCD evidence relevant to Saudi Arabia and were at least publicly available as a report, article, abstract, or data portal were included. Official and nationally representative sources were prioritized for core analysis [5,6,7,8,9,14], while regional, clinical, convenient, and disease-specific studies were kept solely for contextual purposes.

After title/abstract and full-text screening, eligible peer-reviewed Saudi nutrition studies were retained and combined with official Saudi reports, WHO/MoH reports, GASTAT publications, and global public databases. After source consolidation, 16 source-level entries were included in the final evidence map (Supplementary Figure S1).

### 2.6. Harmonization of Data

Data harmonization was performed to enhance consistency among different types of public datasets retrieved from different sources. The original meaning and messages conveyed by the indicator data were maintained during harmonization. Supply-side food availability, self-reported dietary behavior, measurable biomarkers, modeled dietary estimates, and contextual peer-reviewed evidence were the initial categories of sources based on their analytical methods. These categories were not combined statistically because of their different construct representations [1,2,3,4].

### 2.7. Data Extraction

For each included source, the extracted fields included source type, publication or update year, population, age range, sampling design, weighting status (where reported), indicator definition, estimate, denominator or sample size (where available), confidence interval or standard error (where available), exact report table/page or article table, public accessibility, and role in synthesis. Denominators, confidence intervals, and standard errors that were unavailable in the public aggregate source were recorded as not reported rather than estimated.

Since this was a single-author study, independent duplicate screening was not performed for data source screening, duplicate handling, or data extraction. The risk of error was reduced by conducting eligibility screening and data extraction in two passes and rechecking all included estimates against the original source before the final synthesis. The first check was performed in June 2026, while the second check was performed in July 2026 before the manuscript was submitted. A recheck was performed once again before re-submission in August 2026.

### 2.8. Source Appraisal

Sources were appraised using a prespecified descriptive framework rather than a quality score. Appraisal domains included representativeness, recency, measurement validity, public accessibility, indicator comparability, and role in synthesis.

### 2.9. Synthesis and Triangulation

Evidence streams were synthesized descriptively and kept analytically separate. Food availability indicators were interpreted only as supply-side evidence. Self-reported dietary indicators were interpreted as dietary adequacy or behavior according to each source definition. Biomarkers were interpreted as measured biological risk indicators and not as direct dietary outcomes. Triangulation was used to compare the presence, limitations, and interpretive implications of these non-equivalent evidence streams, not to pool estimates or infer individual-level causality [1,9]. For example, fruit and vegetable adequacy was reported as meeting food-group recommendations, consuming five or more servings daily, or meeting CDC-specified fruit and vegetable criteria. These estimates were therefore compared descriptively rather than pooled [6,8,15]. Biomarker indicators retained their original survey definitions. Raised cholesterol, raised glucose, low hemoglobin, and abnormal waist–hip ratio were summarized by age group, where ages were available. No pooling was performed across fasting and nonfasting biochemical measures because their interpretation differs [7,14].

As the considered data sources significantly differed in design, measurement tools, dietary definition, population, and availability of individual-level data, the current study did not consider performing a meta-analysis, regression modeling, or statistical hypothesis testing. In addition, public microdata linking individual dietary intake to biomarkers was not available [3,4,13]. The main analytical approach was triangulation. Food availability indicators were compared against reported dietary adequacy and biomarker-defined risk to identify convergence or discordance across public-data streams. The findings were interpreted at the surveillance level, not as individual-level causal associations [3,4].

## 3. Results

### 3.1. Scoping Yield and Evidence Map

An extensive search identified official national surveys, government statistical publications, worldwide food-supply databases, global dietary modeling platforms, KSA-specific dietary guideline tools, national and regional dietary reports, and biomarker-oriented studies [1,2,5,6,7,8,9,10,11,12,13,14]. Considerable variation was observed in the representativeness and depth of measurement among the data sources, reports, and studies. Table 2 lists the source types, their years of publication, and their role and utilization in the current study.

### 3.2. Core Public-Data Gaps

The most important finding in this study is the variation in data structure among sources. Although the KSA has strong public information on food availability and health outcomes, public individual-level data linking detailed dietary intake to biomarkers are limited [1,6,7,8,9,14]. However, FAOSTAT and GASTAT Food Security Statistics describe food supply, while SHIS 2013 provides the most comprehensive publicly reported national intake data. WHS 2019 provides substantial biomarker details, but the scope of its diet module is much narrower than that of a full nutrition intake survey. Table 3 lists the dietary indicators (domain), the public sources, and their key limitations.

### 3.3. Food Availability Compared with Dietary Adequacy

Food availability statistics for 2024 indicate a significant food supply [9]. Despite this, other parameters reveal low dietary adequacy: according to SHIS 2013, just 5.2% and 7.5% of people met fruit and vegetable intake recommendations, respectively [6,15]. Similarly, according to the GASTAT Health Determinants Statistics 2024, only 10.2% of people aged 15 and above ate five or more servings of fruits and vegetables per day [8]. These indicators describe different surveillance constructs and therefore should be interpreted separately rather than as directly comparable measures (Table 4).

**Table 4:** Food availability, dietary adequacy, and biomarker indicators extracted from public Saudi data sources.

Domain	Variable	Unit	Year	Source	Population	Value	Interpretation
Diet behavior	Fruit consumed days/week	Mean days/week	2005	STEPS	15–64	3.5	Mean; men 3.4, women 3.6
Diet behavior	Fruit consumed 5+ servings/day	%	2005	STEPS	15–64	2.1	Men only extracted from available table
Diet behavior	Vegetable consumed 5+ servings/day	%	2005	STEPS	15–64	2.4	Men only extracted from available table
Diet behavior	Fruit recommendation met	%	2013	SHIS:diet	15+	5.2	Public Health Nutrition paper
Diet behavior	Vegetable recommendation met	%	2013	SHIS:diet	15+	7.5	Public Health Nutrition paper
Diet behavior	Nut recommendation met	%	2013	SHIS:diet	15+	31.4	Public Health Nutrition paper
Diet behavior	Fish recommendation met	%	2013	SHIS:diet	15+	44.7	Public Health Nutrition paper
Diet behavior	CDC fruit+vegetable guideline met	%	2013	SHIS:fruit/veg	15+	2.6	CDC guideline definition
Diet behavior	Fruit+vegetable five or more servings/day	%	2024	GASTAT Health Determinants	15+	10.2	Self-reported National Health Survey
Diet behavior	Fruit+vegetable 1–4 servings/day	%	2024	GASTAT Health Determinants	15+	84.8	Self-reported National Health Survey
Diet behavior	No fruit/vegetable servings/day	%	2024	GASTAT Health Determinants	15+	5.0	Self-reported National Health Survey
Food availability	Rice availability	kg/person/year	2024	GASTAT Food Security	National	52.1	Plant products
Food availability	Date availability	kg/person/year	2024	GASTAT Food Security	National	35.8	Plant products
Food availability	Milk availability	liters/person/year	2024	GASTAT Food Security	National	70.3	Animal products
Food availability	Poultry meat availability	kg/person/year	2024	GASTAT Food Security	National	46.9	Animal products
Food availability	Table egg availability	eggs/person/year	2024	GASTAT Food Security	National	235	Animal products
Food availability	Red meat availability	kg/person/year	2024	GASTAT Food Security	National	13.2	Animal products
Food availability	Onion availability	kg/person/year	2024	GASTAT Food Security	National	20.5	Vegetable product
Food availability	Tomato availability	kg/person/year	2024	GASTAT Food Security	National	19.56	Vegetable product
Biomarker	Hypercholesterolemia	%	2019	WHS Saudi Arabia	15+	42.6	Measured nonfasting cholesterol >5 mmol/L

Panel A shows selected 2024 national per capita food availability indicators from GASTAT Food Security Statistics and should be interpreted as supply-side food-system evidence, not individual intake. Panel B shows selected self-reported dietary adequacy or intake indicators from SHIS 2013 and GASTAT Health Determinants Statistics 2024. Panel C shows selected measured biomarker-defined risk indicators from WHS Saudi Arabia 2019. The panels are separated because the indicators differ in year, population, denominator availability, measurement definition, and construct; they should not be interpreted as pooled or directly comparable estimates.

### 3.4. Biomarker-Defined Risk Remains High

The most recent public biomarker evidence comes from WHS 2019 [7]. Hypercholesterolemia was recorded as prevalent and increased with age. Approximately half of respondents reported low hemoglobin, most commonly in elderly people. The waist-to-hip ratio was abnormally high in all age categories.

The cells show the total percentage of respondents within each age group with abnormal waist–hip ratio, raised random blood glucose, raised serum cholesterol, and low hemoglobin according to WHS 2019 definitions. The denominators differ by indicator and age group and are provided in Supplementary Table S2. Low hemoglobin is reported as a measured indicator and should not be equated with clinically adjudicated anemia unless the original source explicitly defines it as such.

### 3.5. Recent Public Research Adds Important but Partial Evidence

Recent studies give detailed information on beverages, sugar consumption, adolescent dietary behavior, and diet-quality approaches [16,17,18,19,20,21,22]. The 2025 nationwide beverage survey includes current adult beverage data from all 13 regions [17]. The adolescent beverage study measures beverage and SSB intake among Riyadh adolescents [18]. Studies on free-sugar intake among children and fast-food consumption among adolescents reveal significant exposure to sweetened beverages and energy-dense foods [19,20,21]. Though these studies are important academic research initiatives, they cannot replace a national individual-level biomarker-paired food-intake survey (Table 5).

**Table 5:** Recent Saudi studies contributing contextual evidence on beverages, sugar intake, diets of adolescents, and dietary guidance.

Topic	Study/Source	Population	Key Public Value	Analytical Use
Adults’ beverage consumption	Patterns of Beverage Consumption Among Saudi Residents	4385 adults, 13 regions	Sweetened tea 28 mL/day; regular soft drinks 22.1 mL/day	Recent national beverage context
Adolescents’ beverage consumption	BMC Public Health adolescent beverage study	316 Riyadh adolescents aged 15–19	TBC 1702 mL/day; SSB 478 mL/day	Youth SSB context
Children’s sugar intake	Sources of free sugar in Saudi children	Saudi children	Sweetened beverages 27.2 g/day free sugar	Sugar-source context
Adolescents’ fast-food consumption	Western Saudi adolescent study	617 adolescents aged 11–18	28.5% frequent fast food >2 times/week	Diet-quality context
Dietary guidance	Saudi Healthy Plate 2024	Guideline model	Culturally tailored optimized dietary patterns	Comparator standard

## 4. Discussion

**Figure 1:**
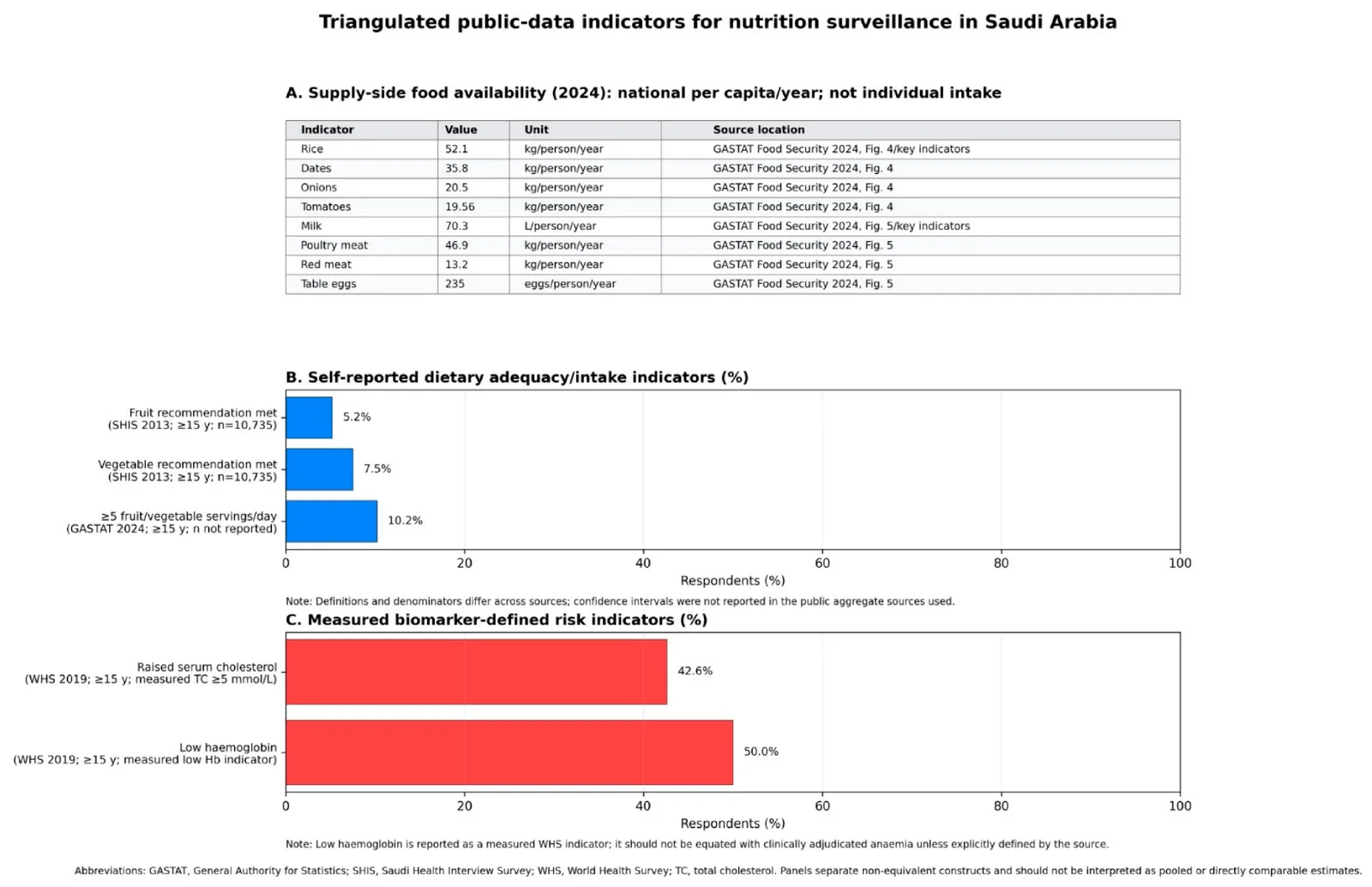
A graphical representation of separate public-data streams relevant to nutrition surveillance in Saudi Arabia.

**Figure 2:**
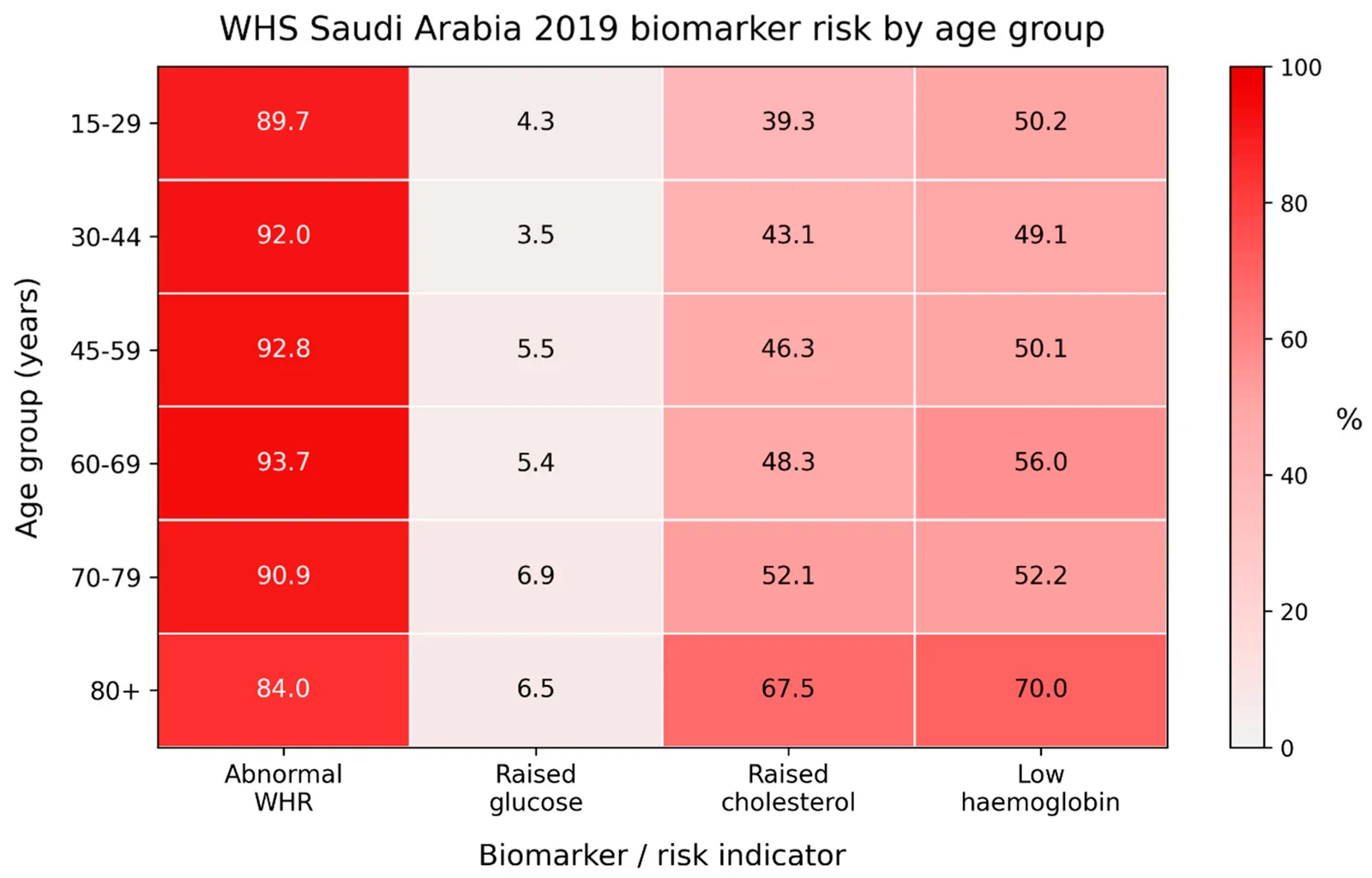
A heat-map representation of biomarker-defined nutritional and metabolic risk by age group in the World Health Survey Saudi Arabia 2019.

This study confirms that Saudi Arabia is not completely lacking in public nutrition information; rather, it has a fragmented evidence ecosystem. Official sources provide useful food availability, health determinant, and biomarker indicators. Peer-reviewed studies provide important details on specific foods, beverages, sugar, dietary habits of children and adolescents, and dietary instruments (Table 5). However, these data streams do not substitute for a current, nationally representative, public individual-level food consumption survey with biomarker linkage [1,2,6,7,8,9,14,16,17,18,19,20,21,22] (Table 1 and Table 2). This distinction has important policy implications. Even if food security and food availability statistics show adequate national supply and improved self-sufficiency for selected products [8,9], a population may still fail to meet healthy dietary recommendations because of preferences, affordability, marketing, food environments, knowledge, culture, time constraints, or differential access [5,23]. Conversely, biomarkers such as low hemoglobin or cholesterol (Figure 1 and Figure 2) are influenced by factors beyond diet alone [3,4,7]. The correct method of interpretation is therefore triangulation, not direct causal equivalence.

As a matter of fact, the principal inference of this study is not that food availability should directly correspond to dietary adequacy or biomarkers, but that current public-data streams represent different and only partially connected dimensions of nutrition surveillance. Food availability statistics describe the national supply, not individual intake; hence, their coexistence with low dietary adequacy indicators or high biomarker-defined risk should not be interpreted as proof of a direct association, as these can be explained by food loss and waste, imports, distribution, affordability, household allocation, dietary preferences, survey error, demographic differences, inflammation, genetics, comorbid disease, medication use, and other non-dietary determinants of biomarkers.

The novelty of the present approach is the explicit separation of supply, intake, and biomarker constructs [1,2,3,4]. This avoids overinterpreting food balance sheets as nutrition intake and biomarkers as dietary outcomes. It also complements Saudi Healthy Plate and NNC model studies by showing where measured public surveillance remains insufficient [10,11,12]. The findings of SHIS 2013 and GASTAT 2024 should not be considered a formal chronological trend because the two reports considerably differ in their indicator definitions and survey techniques. Nevertheless, both sources point in the same substantive direction—only a minority of the population achieves the recommended fruit and vegetable intake [6,8,15]. These observations suggest that determining national dietary adequacy requires integrated surveillance linking dietary intake and biomarkers, rather than demonstrating that national food availability is either sufficient or insufficient.

The biomarker profile raises an additional concern. WHS 2019 reported substantial hypercholesterolemia, low hemoglobin, and abnormal waist–hip ratio, while the PURE-Saudi study and recent dietary research describe unhealthy dietary patterns and pro-inflammatory diets [7,23,24]. These findings do not mean that the observed anomalies are caused by the reported dietary indicators. On the contrary, they show that favorable supply-side statistics can coexist with persistent metabolic and nutritional vulnerability. Recent beverage, free-sugar, fast-food, and adolescent studies show why a national food-wide survey is needed. Their restricted scope and lack of linked biomarkers limit national inference [16,17,18,19,20,21,22]. A repeated survey using standardized 24-h recalls or validated Saudi food-frequency questionnaires, along with biomarker subsamples and public microdata, would permit the direct assessment of dietary adequacy and policy effects [2,3,4,22].

This study relied on KSA official statistics, WHO-aligned surveys, and global databases, as well as the contemporary literature. The strength of this study is that evidence was rated based on its representativeness and the nature of the data [1,2,5,6,7,8,9,10,11,12,13,14,15,16,17,18,19,20,21,22,23,24]. However, a few crucial limitations were encountered during this study. Microdata were not provided in some published reports, and indicators were defined differently from study to study and report to report [3,4]. Also, the possibility of publication bias could not be ruled out, and the descriptive design of the study precludes causal inference at the individual level [3,4]. In addition, a meta-analysis was not considered, as the data sources were not adequately harmonizable in terms of design, population, dietary definitions, biomarker measurement, and availability of variance estimates. Future work could apply meta-analytic methods if more harmonized data become available. A future quantitative synthesis could predefine comparable outcomes, such as fruit and vegetable adequacy, sugar-sweetened beverage intake, anemia, raised glucose, dyslipidemia, obesity, and abnormal waist–hip ratio. Where multiple studies report comparable prevalence estimates with denominators and measures of uncertainty, random-effects meta-analysis could be used to estimate pooled prevalence, with subgroup analyses by sex, age group, nationality, region, and survey year. Meta-regression could further examine whether the survey period, data source type, or measurement method explains between-study heterogeneity.

Despite its limitations, this study provides insight into contemporary data acquisition practices, their availability for independent research, and the possible disadvantage of the variation in data sample criteria at the national level. This study also highlights the need for (i) prioritized periodic national nutrition intake surveys using standard 24-h recalls or validated food frequency questionnaires (FFQs) with pivotal demographic stratification; (ii) inclusion biomarker sub-samples such as hemoglobin, iron indices, HbA1c or fasting glucose, lipids, vitamin D, and anthropometrics; and (iii) anonymously compiled microdata for individual and independent studies.

## 5. Conclusions

Publicly available Saudi nutrition evidence provides useful but non-equivalent information on food availability, dietary adequacy, and biomarker-defined risk. However, current public-data streams represent different and only partially connected dimensions of nutrition surveillance. Food availability and self-sufficiency indicators show increasingly available food supply, but dietary adequacy remains low, and biomarker-defined risk is elevated. This study highlights the need for a national nutrition surveillance policy that links repeated dietary intake assessments, harmonized public reporting, biomarker monitoring, and evaluation of nutrition interventions, such as sugar-sweetened beverage taxation, food labeling, school food standards, and reformulation policies. Also, the findings suggest that clinicians should not infer nutritional status from food availability alone but should combine dietary history, anthropometric assessment, and relevant biomarkers to identify individual nutritional and cardiometabolic risks. The most meaningful next research and policy step is to develop programs for transparent, repeated, individual-level nutrition surveillance linked to biological markers. Until then, food availability data should be interpreted as food-system evidence rather than as a proxy for human nutritional status.

## Figures and Tables

**Table 1:** Search strategy and sources used to identify Saudi nutrition-related public evidence.

Database/Source	Search String	Result/Use
PubMed/PMC/web	Saudi Arabia nutrition intake dietary survey public data Saudi Health Interview Survey diet 2013 fruit vegetables sugar sweetened beverages	Identified SHIS diet paper, fruit/vegetable analysis, SSB analysis
PubMed/PMC/web	Saudi Arabia dietary intake nutrition survey PubMed 2025 adults food frequency questionnaire beverage consumption nationwide	Identified 2025 national adult beverage survey and dietary tools
PubMed/PMC/web	site: pubmed.ncbi.nlm.nih.gov Saudi Arabia dietary intake 2025 adolescents food consumption Saudi	Identified adolescent beverage, adolescent diet/BMI, fast-food papers
PubMed/PMC/web	Saudi Arabia free sugar consumption children nutrient intake PubMed	Identified children’s free-sugar sources and nutrient displacement studies
GASTAT official	Saudi Arabia National Health Survey 2024 Health Determinants Statistics fruit vegetable servings GASTAT	Identified Health Determinants Statistics 2024 PDF/XLSX and news summary
GASTAT official	Saudi Arabia food security statistics 2024 GASTAT per capita availability milk poultry rice dates	Identified Food Security Statistics 2024 PDF and tables
FAO/WHO	Saudi Arabia FAO WHO GIFT individual food consumption data Saudi Arabia	Confirmed FAO/WHO GIFT purpose and lack of directly usable Saudi open IQFC dataset in search results
Global data	Saudi Arabia Global Dietary Database dietary intake age sex Saudi Arabia 2019 GDD	Identified GDD and GNR profile references; use as modeled secondary context

**Table 2:** Evidence map of public Saudi food, nutrition, dietary, and biomarker data sources.

Source ID	Source Type	Title	Year	Analytical Role	Remarks on the Reason of Utilization
S01	Official national survey	WHO STEPS Saudi Arabia NCD Risk Factors Standard Report	2005	Core	STEPS design; national baseline; age–sex tables available
S02	Official national survey	World Health Survey Saudi Arabia (KSAWHS)	2019	Core	Main recent biomarker source; age–sex and region tables
S03	Official statistical publication	GASTAT Health Determinants Statistics Publication	2024	Core	Latest official fruit/vegetable adequacy and obesity estimates
S04	Official statistical publication	GASTAT Food Security Statistics	2024	Core	Latest official Saudi food availability data
S05	Official international data	FAOSTAT Food Balance Sheets	2010–2023	Core	Supply-side data; not individual intake
S06	Academic national survey	Diet in Saudi Arabia: findings from a nationally representative survey (SHIS)	2013	Core	Most detailed public national diet-intake evidence
S07	Academic analysis of SHIS	Fruit and vegetable consumption among adults in Saudi Arabia, 2013	2015	Core/context	Reports very low fruit/vegetable recommendation adherence
S08	Academic analysis of SHIS	Determinants of sugar-sweetened beverage consumption in Saudi Arabia	2022	Context	Useful for SSB sub-analysis from national data
S09	Global database	Global Dietary Database (GDD)	1990–2018/2019	Secondary/context	Useful for external comparison and gap assessment
S10	Global data platform	FAO/WHO GIFT	Current	Gap assessment	Documents scarcity of harmonized Saudi IQFC data
S11	Global burden database	GBD 2019 dietary risk estimates	1990–2019	Secondary/context	Contextual burden estimates, not intake survey
S12	Official advisory/guideline	National Nutrition Committee (SFDA) and Saudi Healthy Plate 2024	2025	Context/comparator	Reference standard for dietary adequacy; not measured intake
S13	Academic index	Saudi Healthy Eating Index (SHEI)	2025	Context	Potential future tool to evaluate adherence
S14	Academic model	Probabilistic Mathematical Model for Dietary Consumption and Exposure Assessment in Saudi Arabia	2024	Important comparator	Shows modeling niche already exists; our novelty should be surveillance discordance
S15	Academic/preprint model	Estimating Dietary Intake and Nutrient Adequacy among Adults in Saudi Arabia: NNC-v2	2025	Context only	Not peer-reviewed as of extraction; use cautiously
S16	Academic national beverage survey	Patterns of Beverage Consumption Among Saudi Residents and Associated Demographic Factors	2025	Context/sensitivity	Current nationwide adult beverage data

**Table 3:** Dietary indicators (domain), public sources, and their role in synthesis.

Data Stream/Source Type	Main Sources	Representativeness	Recency	Measurement Validity	Public Accessibility	Indicator Comparability	Role in Synthesis/Caution
Food availability/food supply	GASTAT Food Security 2024; FAOSTAT Food Balance Sheets	National supply-side statistics; not individual-level	High for 2024 GASTAT; FAOSTAT up to 2023/2024 depending on release	Valid for national availability/supply; not valid as individual intake	Public aggregate tables/portal	Low comparability with intake or biomarkers because construct differs	Use only as food-system availability evidence; do not infer intake or nutritional status
Dietary intake/dietary adequacy	SHIS 2013 diet paper; SHIS fruit/vegetable analysis; GASTAT Health Determinants 2024	SHIS: national household survey; GASTAT: national health statistics; exact designs differ	SHIS is older; GASTAT is recent	Self-reported dietary indicators; definitions differ across sources	Published articles/reports; no public microdata identified	Limited comparability because indicators use different definitions and years	Use to characterize reported dietary adequacy/intake; do not pool without harmonized definitions
Biomarker-defined risk	WHS Saudi Arabia 2019; WHO STEPS 2005	National health examination/survey data where available	Moderate; WHS 2019 is the most recent public biomarker source used	Measured biomarkers, but fasting status and definitions vary	Public report tables; no public microdata identified	Limited comparability with food availability and self-reported intake	Use as biological risk indicators; avoid causal attribution to diet alone
Modeled dietary/nutrient estimates	Global Dietary Database; NNC/NNC-v2; Saudi Healthy Plate/SHEI literature	Modeled or guideline/index-based; not direct measured intake	Recent but depends on source/model	Useful for context and comparison; model uncertainty remains	Public abstracts/articles/portals where available	Moderate to low unless model inputs and definitions align	Use as contextual evidence; not a replacement for measured intake-biomarker data
Contextual Saudi studies	Beverage, SSB, free-sugar, adolescent, fast-food, FFQ validation and diet-quality studies	Often regional, subgroup-specific, or topic-specific	High for recent studies	Variable by design and instrument	Published articles; usually no public microdata	Low for national estimates; useful for interpretation	Use to support interpretation and identify evidence gaps, not to estimate national prevalence

## Data Availability

No new data were generated; all data were derived from publicly available sources cited in the manuscript and Supplementary Tables.

## Supplementary Materials

The following supporting information can be downloaded at: https://www.mdpi.com/article/10.3390/nu18172888/s1, **Supplementary Figure S1. PRISMA-ScR flow diagram for source identification and selection.** The diagram summarizes records identified, duplicates removed, records screened, full-text or source-level assessments, exclusions with reasons, and the final source-level entries included in the scoping evidence map. **Supplementary Table S1. Search strategy and source-documentation matrix.** This table provides the final search documentation, including database/source, search date, review period, search terms or Boolean strings, retrieval approach, record counts, and source-selection notes to support replication or extension of the scoping search. **Supplementary Table S2. Extracted indicators and source-level methodological details.** This table summarizes the extracted food-availability, dietary-adequacy, and biomarker-defined indicators, including source, year, population, denominator where available, sampling/weighting information, indicator definitions, confidence intervals or standard errors, source location, and interpretation cautions.
