# Supplementary material for: Assessment of Nutritional Risk Representations in Publicly Available Data in Saudi Arabia: A Triangulated Scoping Study
Source: Nutrients. 2026 Sep 3;18(17):2888. doi: 10.3390/nu18172888 (PMC13567205; doi:10.3390/nu18172888)
Supplement: Supplementary file 1 [file nutrients-18-02888-s001.zip › nutrients-4449381-supplementary Figure S1.pdf]

Supplementary Materials

Assessment of Nutritional Risk Representations in Publicly Available Data in Saudi Arabia: A Triangulated Scoping Study

Reham I. Alagal

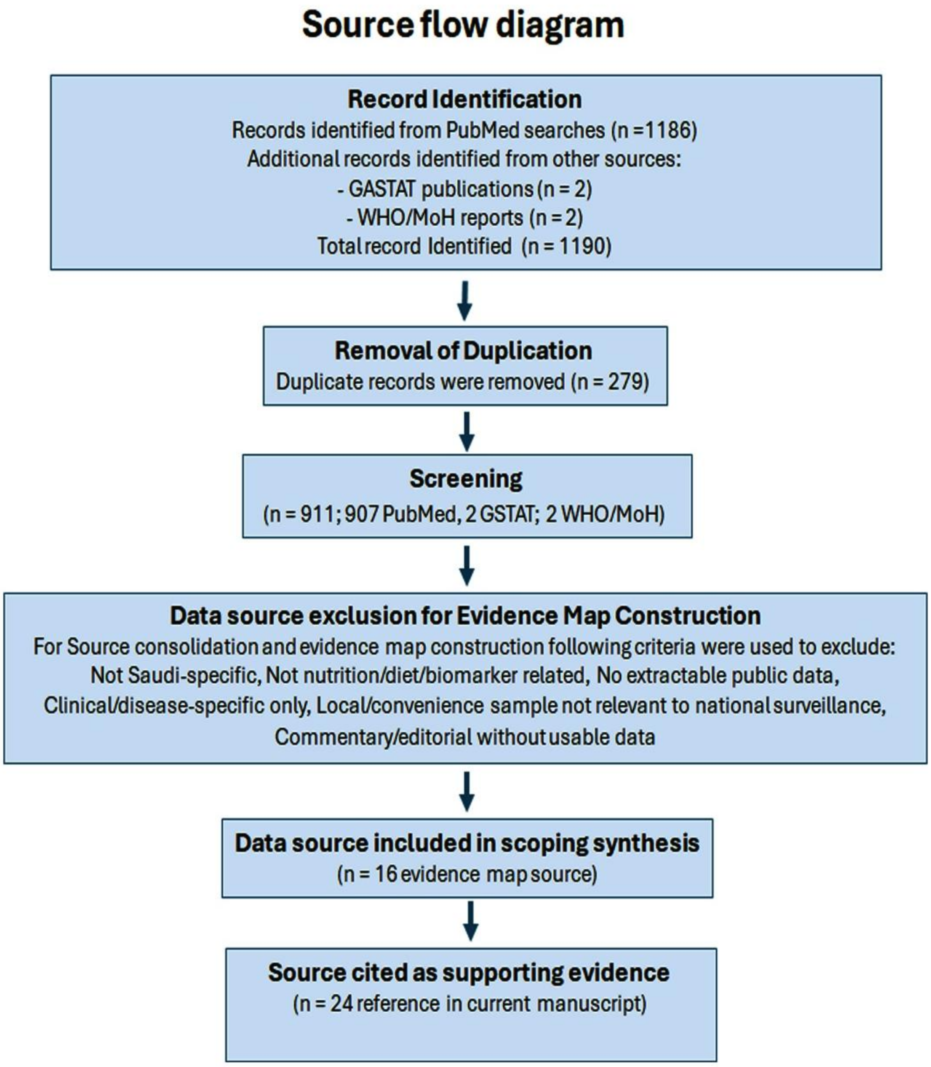

Figure S1.
